# Favorable glycemic response after pancreatoduodenectomy in both patients with pancreatic cancer and patients with non-pancreatic cancer

**DOI:** 10.1097/MD.0000000000010590

**Published:** 2018-05-04

**Authors:** Seo Young Sohn, Eun Kyung Lee, Sung-Sik Han, You Jin Lee, Yul Hwangbo, Young Hwa Kang, Seung Duk Lee, Seong Hoon Kim, Sang Myung Woo, Woo Jin Lee, Eun Kyung Hong, Sang-Jae Park

**Affiliations:** a Department of Internal Medicine, National Cancer Center; b Division of Endocrinology and Metabolism, Department of Internal Medicine, Myongji Hospital; c Department of Surgery; d Department of Pathology, National Cancer Center, Goyang, Gyeonggi, Korea.

**Keywords:** diabetes mellitus, glucose metabolism, pancreatic cancer, pancreatoduodenectomy, periampullary cancer

## Abstract

Diabetes mellitus (DM) is prevalent in patients with pancreatic cancer and tends to improve after tumor resection. However, the glycemic response of non-pancreatic cancer patients after surgery has not been examined in detail. We aimed to investigate the changes in glucose metabolism in patients with pancreatic cancer or non-pancreatic cancer after pancreatoduodenectomy (PD).

We prospectively enrolled 48 patients with pancreatic cancer and 56 patients with non-pancreatic cancer, who underwent PD. Glucose metabolism was assessed with fasting glucose, glycated hemoglobin (HbA1c), plasma C-peptide and insulin, quantitative insulin check index (QUICKI), and a homeostatic model assessment of insulin resistance (HOMA-IR) and β cell (HOMA-β) before surgery and 6 months after surgery. Patients were divided into 2 groups: “improved” and “worsened” postoperative glycemic response, according to the changes in HbA1c and anti-diabetic medication. New-onset DM was defined as diagnosis of DM ≤ 2 years before PD, and cases with DM diagnosis >2 years preceding PD were described as long-standing DM.

After PD, insulin resistance (IR), as measured by insulin, HOMA-IR and QUICKI, improved significantly, although C-peptide and HOMA-β decreased. At 6 months after PD, new-onset DM patients showed improved glycemic control in both pancreatic cancer patients (75%) and non-pancreatic cancer patients (63%). Multivariate analysis showed that long-standing DM was a significant predictor for worsening glucose control (odds ratio = 4.01, *P* = .017).

Favorable glycemic control was frequently observed in both pancreatic cancer and non-pancreatic cancer after PD. PD seems to contribute improved glucose control through the decreased IR. New-onset DM showed better glycemic control than long-standing DM.

## 1 Introduction

Diabetes mellitus (DM) is prevalent in patients with pancreatic cancer and is often exacerbated in patients following a diagnosis of pancreatic cancer.^[1,2]^ There is a close temporal relationship between the development of DM and the diagnosis of pancreatic cancer. Reports indicate that DM is prevalent in up to two-third of pancreatic cancer patients,^[2,3]^ with pancreatic cancer-associated diabetes generally occurring during the 2 years preceding the diagnosis of cancer.^[3–5]^ Experimental results suggest that new-onset DM in pancreatic cancer patients is a paraneoplastic manifestation.^[4,6]^

Pancreatoduodenectomy (PD), or pylorus-preserving PD, is a standard operation for periampullary malignancies that removes almost 40% of the whole pancreas and the adjacent organs, such as the gallbladder, duodenum, and distal bile duct. Because it is one of the most radical types of gastroenterological surgery, patients who undergo this procedure frequently experience endocrine and exocrine pancreatic insufficiency.^[7]^ However, several studies have reported that the deterioration in glucose metabolism is often improved following tumor resection in patients with pancreatic cancer, despite a significant reduction in pancreatic cell mass due to surgery.^[4,8–11]^ Most of these studies have focused on changes in diabetes status, including whether the diabetes developed newly after surgery or whether glycemic control improved or worsened in diabetic patients. However, there are sparse studies investigating the detailed mechanism of altered glucose metabolism, such as the changes in β-cell function or insulin resistance (IR) after PD.^[8]^ Moreover, it remains unclear whether glucose metabolism also improves after PD in patients with non-pancreatic neoplasms.

In this study, we prospectively investigated the changes in glucose metabolism in patients undergoing PD for periampullary cancer. We also aimed to identify the predictive factors for worsening glucose metabolism after PD.

## 2 Methods

### 2.1 Subjects and procedure

Between April 2011 and December 2013, 104 consecutive patients (age, mean ± standard deviation [SD] 64.8 ± 9.6 years; 69 male patients) undergoing PD in National Cancer Center, Korea were prospectively enrolled. Of these, 48 patients were diagnosed with pancreatic cancer and 56 patients with a final pathology, other than pancreatic adenocarcinoma. These were classified as the non-pancreatic cancer group and included 29 patients with carcinomas of the ampulla of Vater, 26 with distal common bile duct cancers, and 1 with duodenal cancers. Patients with pre-existing chronic pancreatitis only, on the pathology report, were excluded from the analysis.

The parameters related to glucose tolerance were assessed before surgery and 6 months after surgery by measuring the levels of fasting blood glucose (FBG), glycated hemoglobin (HbA1c), fasting C-peptide, and fasting insulin. HbA1c was assessed using high-performance liquid chromatography, with a nationally standardized program-certified method. Plasma C-peptide (Izotop, Budapest, Hungary) and insulin (Roche, Mannheim, Germany) levels were measured in duplicate using an immunoradiometric assay. C-peptide levels were used to assess endogenous insulin secretion, which has been recognized as a more stable and accurate marker of insulin secretion than plasma insulin levels.^[12]^ IR was assessed by fasting insulin levels^[13]^ and homeostatic model assessment of insulin resistance (HOMA-IR)^[14]^ and quantitative insulin sensitivity check index (QUICKI). To analyze HOMA-IR, the following calculation was used: fasting insulin concentration (μL/mL) × FBG (mg/dL)/405. QUICKI was calculated as follows^[15]^: 1/[log (fasting insulin, μL/mL) + log (FBG, mg/dL)]. To evaluate the insulin secretory function of pancreatic β-cells, HOMA-β was used, and calculated as follows^[14]^: 360 × fasting insulin/(FBG-63). The study protocol was approved by the Institutional Review Board of National Cancer Center (NCC2015-0120) and written informed consent was obtained from all patients.

### 2.2 Definition of glucose tolerance

Patients who previously received antidiabetic medication, were classified as having DM. Among patients not reporting treatment for DM, the definition of DM was based on the American Diabetes Association criteria during preoperative evaluation: FBG ≥126 mg/dL, or HbA1c ≥6.5%. Impaired glucose tolerance (IGT) was defined as having an FBG between 100 and 125 mg/dL, or an HbA1c between 5.7% and 6.4%. Normal glucose tolerance (NGT) was defined as having an FBG <100 mg/dL, and an HbA1c of <5.7%.

The duration of DM was defined as the period between the onset of DM and the time when PD was performed. New-onset DM was defined as a diagnosis of DM <2 years before PD, while DM diagnosed ≥2 years preceding PD was described as long-standing.

Postoperative glycemic control 6 months after surgery was categorized as “worsened” or “improved,” according to the change in the HbA1c levels or in the antidiabetic medication dosage. In preoperative diabetic patients, the “worsened group” was defined by the increase in the dose of antidiabetic medication after PD or increase in the HbA1c, compared with the preoperative dose. In preoperative non-diabetic patients, the appearance or aggravation of glycemic intolerance (from NGT to IGT or overt diabetes, or from IGT to DM) was a characteristic of the worsened group. In preoperative diabetic patients, the “improved group” was defined as having a decrease in the dose of antidiabetic medication based on HbA1c levels. In preoperative non-diabetic patients, the improved group was defined as a return to NGT from IGT or decreased HbA1c levels relative to the baseline value.

### 2.3 Statistical analysis

The data are presented as the mean ± SD or the median with an interquartile range (25th–75th percentile). The paired *t* test or the Wilcoxon signed-rank test was used to compare all measurements at baseline and 6 months after surgery. Logistic regression analysis was used to identify independent predictors of worsening status of glucose control, at 6 months postoperatively. Assuming that patients with higher preoperative HbA1c levels might have easily improved to better glycemic control after PD, we performed a propensity score matching analysis to adjust baseline HbA1c differences between pancreatic cancer and non-pancreatic cancer patients, in the logistic regression analysis. All statistical analyses were performed using the STATA software version 11 (Stata Corp, College Station, TX). The *P* value for statistical significance was defined as *P* < .05.

## 3 Results

### 3.1 Baseline demographics and pre-operative glycemic variables

Baseline characteristics and preoperative glycemic variables of patients are described in Table 1. The pancreatic cancer and non-pancreatic cancer group were compared and no differences were found in age, sex ratio, and preoperative body mass index (BMI). Before surgery, there were 68 diabetic patients in this study, including 39 diabetic patients in pancreatic cancer group and 29 diabetic patients in non-pancreatic cancer group. Preoperative DM was more prevalent in the pancreatic cancer group (81% vs 52%, *P* = .002), with HbA1c and FBG being higher than in the non-pancreatic cancer group. Among the diabetic patients, new-onset DM was identified in 24 (62%) of 39 in the pancreatic cancer patients and in 16 (55%) of 29 non-pancreatic cancer patients, although this difference was not significant (*P* = .598).

**Table 1:**
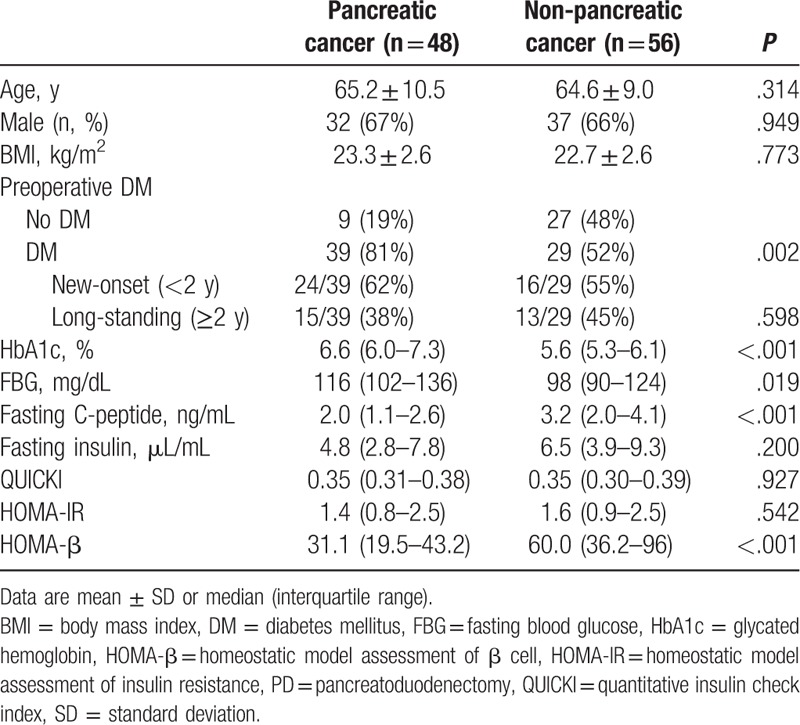
Baseline characteristics of patients before PD.

Fasting C-peptide levels and HOMA-β values were lower in the pancreatic cancer group (C-peptide, 2.0 vs 3.2 ng/mL, *P* < .001; HOMA-β, 31.1% vs 60.0%, *P* < .001) compared to the non-pancreatic cancer group. There was no difference in fasting insulin levels, QUICKI, and HOMA-IR values between the groups.

### 3.2 Change of glucose metabolism after PD

Glucose metabolism was followed up for at least 6 months after PD (Table 2). After surgery, pancreatic cancer patients exhibited a modest improvement in HbA1c 6 months postoperatively, compared with baseline values, with a 0.5% decrease in the median HbA1c value; however, the median HbA1c levels significantly increased in the group of non-pancreatic cancer patients, from 5.6% at baseline and to 6.1% at 6 months, following surgery. In both groups, FBG levels after PD did not change significantly from the baseline. Insulin secretary function, assessed by fasting C-peptide and HOMA-β levels, decreased in both groups. IR, assessed by fasting insulin levels, HOMA-IR and QUICKI significantly improved after surgery in both groups.

**Table 2:**
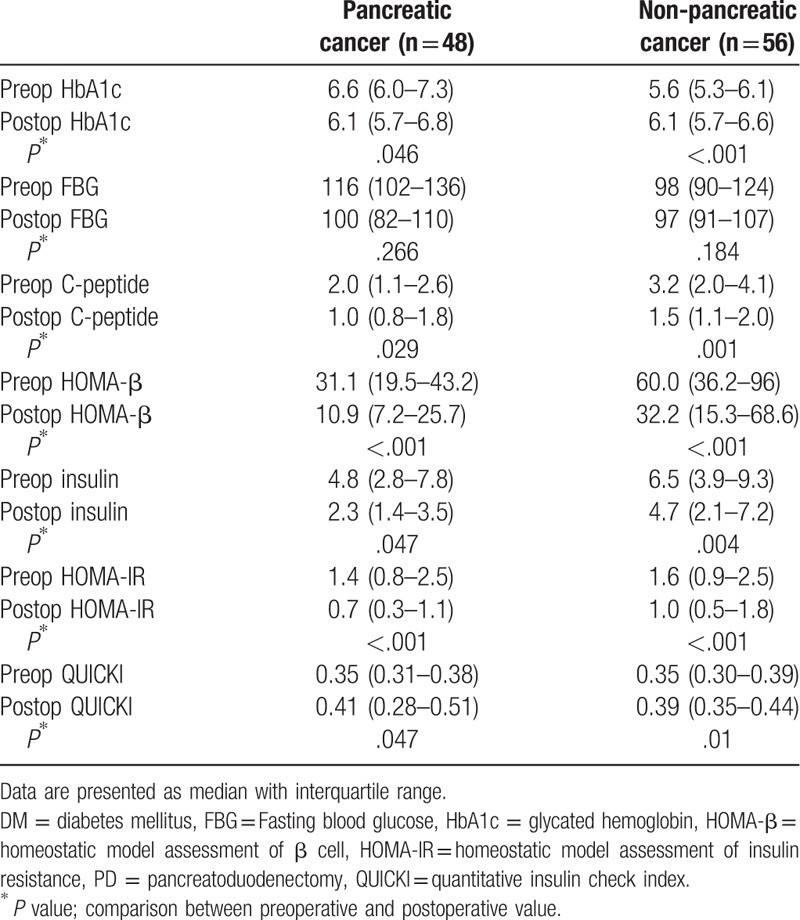
Changes in the parameters of glucose metabolism after PD.

**Figure 1:**
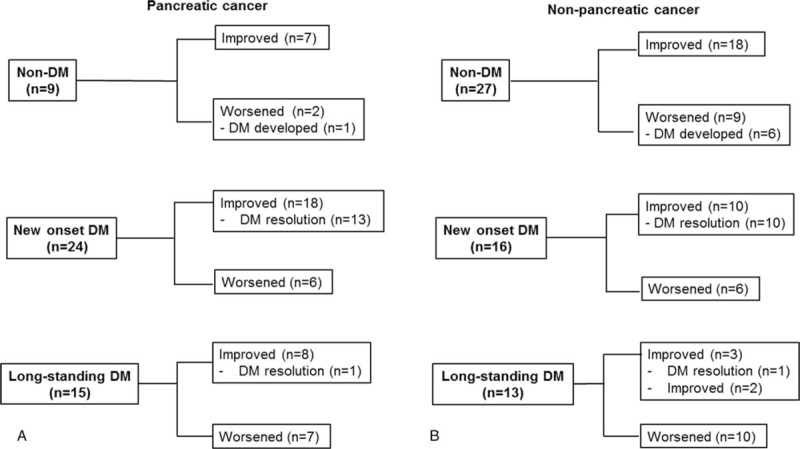
Change of glucose metabolism after surgery according to preoperative DM status. (A) Change of glucose metabolism in pancreatic cancer patients. (B) Change of glucose metabolism in non-pancreatic cancer patients. DM = diabetes mellitus.

Following surgery, patients were stratified according to preoperative DM status (Fig. 1). Of the 36 preoperative non-diabetic patients, 7 patients (19.4%) progressed to DM. DM was postoperatively developed in 1 out of 9 patients with pancreatic cancer and in 6 out of 27 patients with non-pancreatic cancer (11.1% vs 22.2%, *P* = 0.47). Of the 68 preoperative diabetic patients, DM resolved in 25 patients (36.8%). New-onset DM resolved after PD in 54.2% and 62.5% of patients in the pancreatic cancer group and non-pancreatic cancer, respectively (*P* = .42). Resolution of long-standing DM after PD was rarely observed in either group (6.2% in the pancreatic cancer group vs 7.6% in the non-pancreatic cancer group, *P* = .74).

### 3.3 Comparison of characteristics between the groups in terms of improved and worsened glucose metabolism at 6 months after PD

To identify the favorable parameters of glycemic response after PD, the glycemic control status was categorized into 2 groups, as “improved” or “worsened” (Table 3). Improved glycemic control was observed in 66 (63%) patients, while in 38 patients (37%) control worsened. There were no differences between the 2 groups in the preoperat*i*ve glycemic variables, proportion of pancreatic cancer, sex ratio, postoperative weight loss, and perioperative chemotherapy and radiotherapy. However, preoperative DM status was significantly different between groups. Worsened response group had higher rates of long-standing DM than improved response group (42.0% in the worsened group vs 18.2% in the improved group, *P* = .021).

**Table 3:**
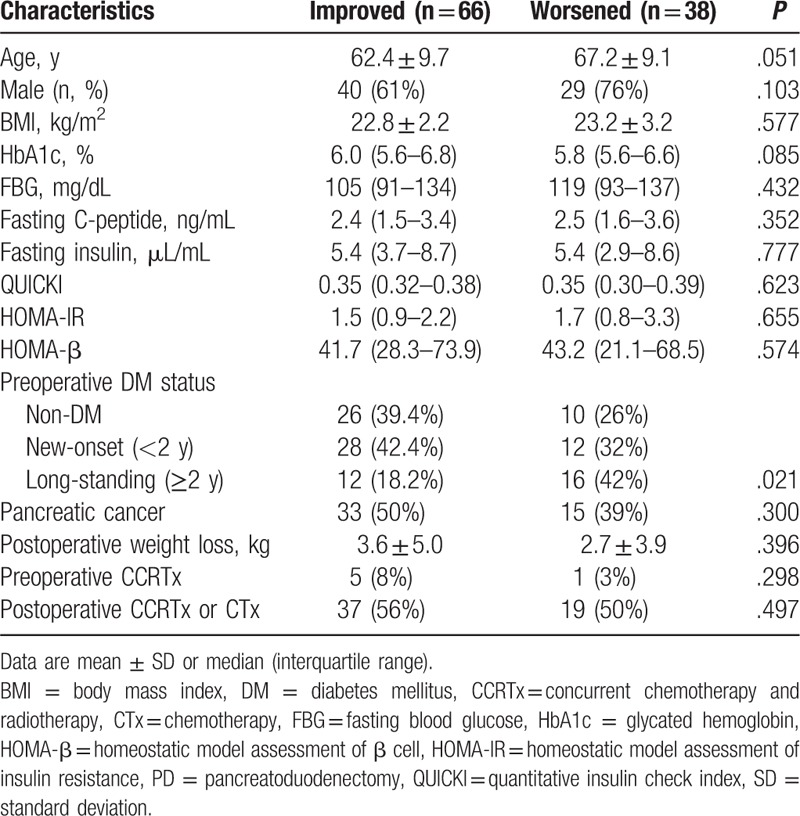
Comparison of characteristics between improved and worsened group at 6 months after PD.

**Figure 2:**
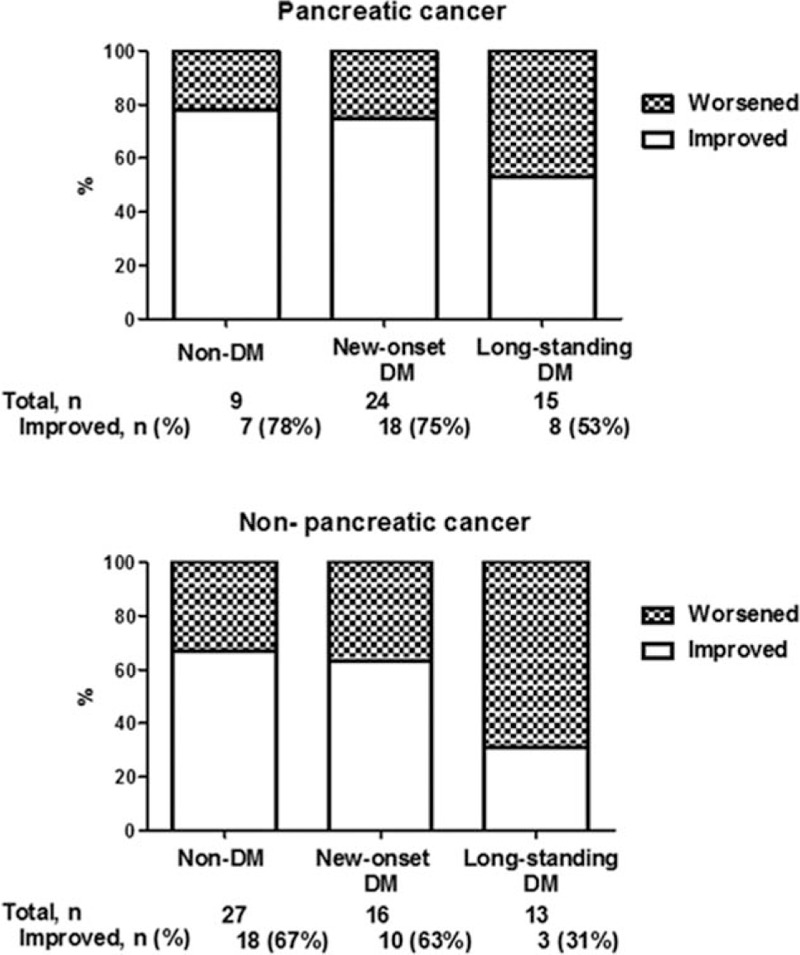
Proportion of improved glucose metabolism at 6 months after surgery. (A) Proportion of improved glucose metabolism in pancreatic cancer patients. (B) Proportion of improved glucose metabolism in non-pancreatic cancer patients.

Multiple logistic regression analysis revealed that long-standing DM (odds ratio [OR]: 4.01, 95% confidence interval [CI]: 1.29–12.50, *P* = .017) was a significant predictor for worsened glucose control after PD (Table 4). Even after propensity score matching for preoperative HbA1c, patients with long-standing DM demonstrated worsened glucose control after PD (OR 5.55, 95% CI 1.23–24.40, *P* = .025). The origin of periampullary tumors was not a significant predictor for worsened glucose control in the multiple regression analysis (Fig. 2).

**Table 4:**
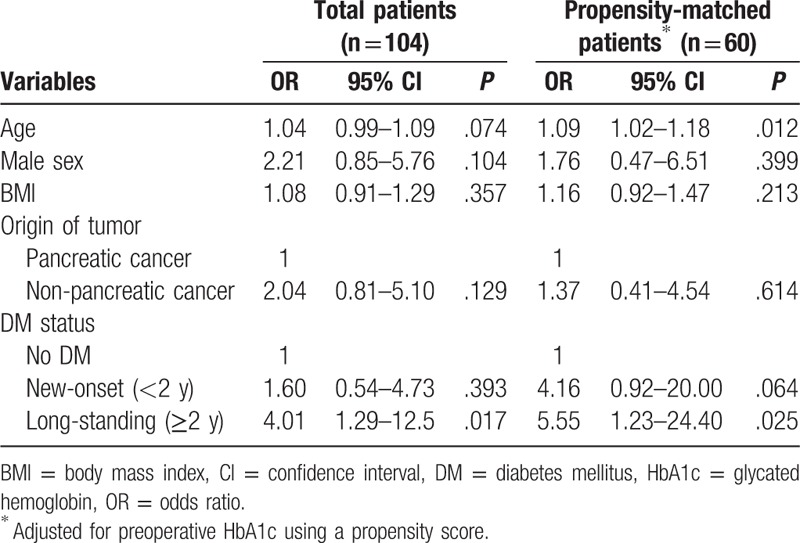
Multiple logistic regression analysis to predict worsening glucose control at 6 months after surgery.

### 3.4 Change of glucose metabolism after PD according to the onset of pre-operative DM

Because new-onset DM showed better glycemic control than long-standing DM after PD (Tables 3 and 4), change in the parameters related with glucose metabolism was re-evaluated according to the onset of pre-operative DM (Table 5). In new-onset DM, FBG level after PD was significantly decreased (*P* = .002), whereas it was increased in long-standing DM (*P* = .018). Insulin secretary function, assessed by fasting C-peptide, decreased in new-onset DM patients and HOMA-β levels decreased in long-standing DM patients. IR, assessed by fasting insulin levels, HOMA-IR and QUICKI, significantly improved after surgery only in the new-onset DM patients.

**Table 5:**
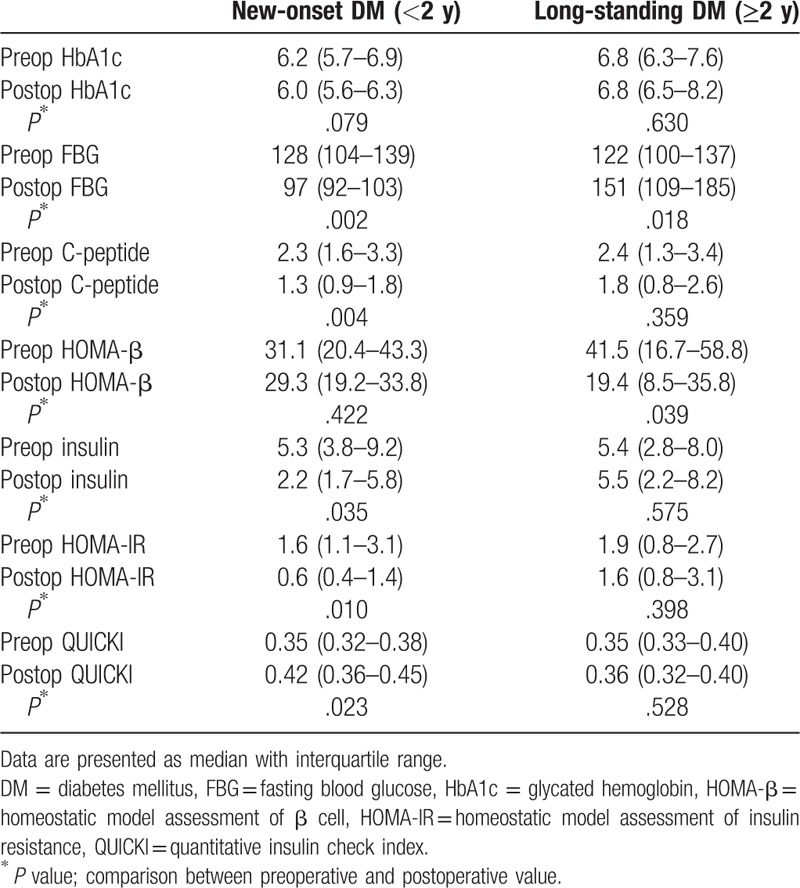
Changes in the parameters of glucose metabolism after surgery according to the onset of preoperative DM.

## 4 Discussion

Our findings demonstrate that newly detected diabetic patients had improved glycemic control after PD, regardless of tumor origin. In multivariate analysis, long-standing DM was a significant risk factor of worsened glucose metabolism in patients undergoing PD, even after propensity score matching for baseline HbA1c levels, long-standing DM was significant predictor for worsening glucose control. Following PD, glycemic variables for IR was improved in both pancreatic cancer and non-pancreatic cancer patients.

In the present study, the prevalence of all DM and new-onset DM was 81% and 62% in 48 pancreatic cancer patients, respectively, which were higher than the general population in Korea.^[16,17]^ Previous studies have reported that more than half of pancreatic cancer patients have DM or hyperglycemia, and the onset of DM typically occurs 2 years before pancreatic cancer diagnosis in 50% to 80% of patients.^[3–5]^ The high prevalence of DM in pancreatic cancer patients and its close temporal association with cancer diagnosis suggests that hyperglycemia is induced by the development of the tumor itself. There are several hypotheses about the occurrence of hyperglycemia in pancreatic cancer. The most persuasive explanation for the frequent occurrence of DM in conjunction with pancreatic cancer is as a paraneoplastic condition caused by tumor-secreted products,^[6]^ such as adrenomedullin.^[18]^

The development of pancreatic DM after pancreatic resection has been of great concern, because surgical resection of the pancreatic parenchyma can cause significant β-cell loss. The incidence of DM varies between 20% and 50% of patients who undergo PD, independently of pre-existing DM.^[19]^ Recent reports have indicated that glucose metabolism improves after PD.^[4,9–11]^ Ohtsuka et al^[8]^ performed an oral glucose tolerance test (OGTT) in 17 patients with periampullary tumors and showed that postoperative levels of plasma glucose and insulin, as well as IR, improved, although β-cell function did not change after surgery. The study examined the mechanism of perioperative changes in glucose metabolism, with some limitations: the number of subjects was insufficient to yield a conclusive result and there was no analysis according to tumor origin. A study by Sato et al^[10]^ also examined glucose tolerance after PD, comparing 11 patients with pancreatic neoplasm and 6 with non-neoplastic lesions, and found a better response in pancreatic cancer patients. These results suggested that there were different glycemic responses following PD, depending on tumor origin. However, the number of subjects was also small and biochemical markers related to glucose mechanism were not analyzed. Specifically, HbA1c levels were not evaluated in these studies.^[8,11,20]^ In our study, glucose metabolism was assessed in a larger population and the glycemic response was analyzed by HbA1c and plasma glucose levels. The results indicate that DM was developed postoperatively in 18% of non-diabetic patients and pre-existing resolved in 36.8% of patients. In addition, IR, as measured by insulin levels, HOMA-IR and QUICKI significantly improved after PD; however, insulin secretion decreased postoperatively.

Furthermore, whether the change in glucose tolerance is a unique feature of pancreatic cancer remains controversial. Our study indicates that improved IR and decreased insulin secretion were consistently observed in both patients with pancreatic cancer and those with non-pancreatic cancer. It suggested that anatomical changes following PD may play a role in the improvement of glucose metabolism. A recent study by Wu et al^[9]^ reported similar results: resolution of new-onset DM after PD was observed in 41% (9 of 22) of patients with pancreatic cancer and in 63% (12 of 19) of patients undergoing surgery for diseases other than pancreatic cancer. However, HbA1c was not included in the analysis and resolution was defined using fasting glucose levels. Menge et al^[20]^ also reported that post-challenge glucose concentrations were immediately improved after pancreatic head resection in patients with pancreatic cancer or non-malignant pancreatic disease. Litwin et al^[11]^ reported a decrease in average fasting glucose levels during and after an OGTT in patients with pancreatic cancer, and an increase in patients with chronic pancreatitis, after PD. Based on our data and previous results, we can postulate that tumor resection decreases insulin secretion, however, it seems to improve IR in both pancreatic cancer and non-pancreatic cancer patients.

Multivariate analysis found that the risk factor for worsened glucose control after PD was long-standing DM. By using the independent *t* test and adjusting for baseline HbA1c, only long-standing DM was associated with worsened glucose control after PD (18% in the improved group vs 42% in the worsened group, *P* = 0.021). In addition, IR, assessed by fasting insulin levels, HOMA-IR and QUICKI, significantly improved after surgery only in the new-onset DM patients, but not in the long-standing DM patients. As for the reversibility of diabetes, several studies have shown that a shorter duration of diabetes has a greater chance of diabetes remission after bariatric surgery.^[21,22]^ This observation suggests that bariatric surgery could lead to remission of diabetes prior to irreversible β-cell failure. The route for food following bariatric surgery is similar to that after PD, as food bypasses the duodenum and enters directly into the distal jejunum. Both PD and bariatric surgery can shunt food past the duodenum, which results in the rapid delivery of nutrients to the distal intestine. This theoretically enhances the release of glucagon-like peptide-1, which stimulates insulin secretion.^[23]^ Another possible explanation is that the early relief of tumor-induced pancreatic duct obstruction and fibrosis of the adjacent parenchyma by PD can preserve a relatively large amount of functional pancreatic tissue in new-onset diabetes, regardless of the origin of the periampullary cancer. This is corroborated by our data, showing that new-onset DM improved after surgery, both in the pancreatic cancer and non-pancreatic cancer group. Therefore, we can conclude that PD may contribute to an improvement in glucose control.

Our study has some limitations. First, stimulated insulin secretion was not evaluated, although these may have reflected β-cell function more accurately than single measurements. However, the HOMA index, used in this study, has been widely used to assess β-cell function^[24,25]^ and a single measurement of blood C-peptide levels can be a practical test to perform during follow-up. Second, long-term glucose metabolism was not evaluated because glucose metabolism was assessed for a relatively short period. Further studies are needed to provide the long-term outcome.

In conclusion, glucose intolerance was prevalent in patients with periampullary cancer and considerably improved in patients with pancreatic cancer after PD. A more favorable glycemic response was observed in patients with new-onset DM compared with patients with long-standing DM, in cases of pancreatic and non-pancreatic cancer. Finally, although the volume of pancreatic parenchyma and secretory function were reduced, PD might contribute to the amelioration of glucose control in patients through the improvement of IR.

## Author contributions

**Formal analysis:** Seo Young Sohn.

**Investigation:** Seo Young Sohn, Eun Kyung Lee.

**Writing – original draft:** Seo Young Sohn.

**Writing – review & editing:** Seo Young Sohn, Eun Kyung Lee, Sung-sik Han.

**Conceptualization:** Eun Kyung Lee, You Jin Lee, Woo Jin Lee, Sung-sik Han.

**Funding acquisition:** Eun Kyung Lee, You Jin Lee.

**Project administration:** Eun Kyung Lee, Woo Jin Lee.

**Methodology:** Yul Hwangbo, Seung Duk Lee, Seong Hoon Kim, Sang Myung Woo.

**Resources:** Yul Hwangbo, Seong Hoon Kim, Sang Myung Woo.

**Data curation:** Young Hwa Kang.

**Validation:** Eun Kyung Hong, Sung-sik Han.

**Supervision:** Sang Jae Park, Sung-sik Han.

## References

[1] Noy A Bilezikian JP Clinical review 63: Diabetes and pancreatic cancer: clues to the early diagnosis of pancreatic malignancy. J Clin Endocrinol Metab 1994;79:1223–31. 7962312 10.1210/jcem.79.5.7962312

[2] Wang F Herrington M Larsson J The relationship between diabetes and pancreatic cancer. Mol Cancer 2003;2:4. 12556242 10.1186/1476-4598-2-4 PMC149418

[3] Aggarwal G Kamada P Chari ST Prevalence of diabetes mellitus in pancreatic cancer compared to common cancers. Pancreas 2013;42:198–201. 23000893 10.1097/MPA.0b013e3182592c96 PMC3896296

[4] Pannala R Leirness JB Bamlet WR Prevalence and clinical profile of pancreatic cancer-associated diabetes mellitus. Gastroenterology 2008;134:981–7. 18395079 10.1053/j.gastro.2008.01.039 PMC2323514

[5] Li D Diabetes and pancreatic cancer. Mol Carcinog 2012;51:64–74. 22162232 10.1002/mc.20771 PMC3238796

[6] Sah RP Nagpal SJ Mukhopadhyay D New insights into pancreatic cancer-induced paraneoplastic diabetes. Nat Rev Gastroenterol Hepatol 2013;10:423–33. 23528347 10.1038/nrgastro.2013.49 PMC3932322

[7] Jang JY Kim SW Park SJ Comparison of the functional outcome after pylorus-preserving pancreatoduodenectomy: pancreatogastrostomy and pancreatojejunostomy. World J Surg 2002;26:366–71. 11865376 10.1007/s00268-001-0234-x

[8] Ohtsuka T Kitahara K Kohya N Improvement of glucose metabolism after a pancreatoduodenectomy. Pancreas 2009;38:700–5. 19506534 10.1097/MPA.0b013e3181a7c916

[9] Wu JM Kuo TC Yang CY Resolution of diabetes after pancreaticoduodenectomy in patients with and without pancreatic ductal cell adenocarcinoma. Ann Surg Oncol 2013;20:242–9. 22864799 10.1245/s10434-012-2577-y

[10] Sato N Yamaguchi K Yokohata K Changes in pancreatic function after pancreatoduodenectomy. Am J Surg 1998;176:59–61. 9683135 10.1016/s0002-9610(98)00105-6

[11] Litwin J Dobrowolski S Orlowska-Kunikowska E Changes in glucose metabolism after Kausch-Whipple pancreatectomy in pancreatic cancer and chronic pancreatitis patients. Pancreas 2008;36:26–30. 18192877 10.1097/mpa.0b013e318137aa61

[12] Rubenstein AH Pottenger LA Mako M The metabolism of proinsulin and insulin by the liver. J Clin Invest 1972;51:912–21. 5014618 10.1172/JCI106886 PMC302205

[13] Hanson RL Pratley RE Bogardus C Evaluation of simple indices of insulin sensitivity and insulin secretion for use in epidemiologic studies. Am J Epidemiol 2000;151:190–8. 10645822 10.1093/oxfordjournals.aje.a010187

[14] Katsuki A Sumida Y Gabazza EC Homeostasis model assessment is a reliable indicator of insulin resistance during follow-up of patients with type 2 diabetes. Diabetes Care 2001;24:362–5. 11213893 10.2337/diacare.24.2.362

[15] Chen H Sullivan G Quon MJ Assessing the predictive accuracy of QUICKI as a surrogate index for insulin sensitivity using a calibration model. Diabetes 2005;54:1914–25. 15983190 10.2337/diabetes.54.7.1914

[16] Park Ie B Kim J Kim DJ Task Force Team for Basic Statistical Study of Korean Diabetes Mellitus of Korean Diabetes Association. Diabetes epidemics in Korea: reappraise nationwide survey of diabetes “diabetes in Korea 2007”. Diabetes Metab J 2013;37:233–9. 23991400 10.4093/dmj.2013.37.4.233 PMC3753487

[17] Ha KH Kim DJ Trends in the diabetes epidemic in Korea. Endocrinol Metab (Seoul) 2015;30:142–6. 26194073 10.3803/EnM.2015.30.2.142 PMC4508257

[18] Aggarwal G Ramachandran V Javeed N Adrenomedullin is up-regulated in patients with pancreatic cancer and causes insulin resistance in beta cells and mice. Gastroenterology 2012;143:1510.e1–7.e1. 22960655 10.1053/j.gastro.2012.08.044 PMC3787599

[19] Stone WM Sarr MG Nagorney DM Chronic pancreatitis. Results of Whipple's resection and total pancreatectomy. Arch Surg 1988;123:815–9. 3382346 10.1001/archsurg.1988.01400310029004

[20] Menge BA Schrader H Breuer TG Metabolic consequences of a 50% partial pancreatectomy in humans. Diabetologia 2009;52:306–17. 19037627 10.1007/s00125-008-1219-1

[21] Dixon JB Dixon AF O’Brien PE Improvements in insulin sensitivity and beta-cell function (HOMA) with weight loss in the severely obese. Homeostatic model assessment. Diabet Med 2003;20:127–34. 12581264 10.1046/j.1464-5491.2003.00889.x

[22] Hall TC Pellen MG Sedman PC Preoperative factors predicting remission of type 2 diabetes mellitus after Roux-en-Y gastric bypass surgery for obesity. Obes Surg 2010;20:1245–50. 20524158 10.1007/s11695-010-0198-8

[23] Orskov C Glucagon-like peptide-1, a new hormone of the entero-insular axis. Diabetologia 1992;35:701–11. 1324859

[24] Kawaguchi T Ide T Taniguchi E Clearance of HCV improves insulin resistance, beta-cell function, and hepatic expression of insulin receptor substrate 1 and 2. Am J Gastroenterol 2007;102:570–6. 17222321 10.1111/j.1572-0241.2006.01038.x

[25] Vashum KP McEvoy M Milton AH Is serum zinc associated with pancreatic beta cell function and insulin sensitivity in pre-diabetic and normal individuals? Findings from the Hunter Community Study. PLoS One 2014;9:e83944. 24416185 10.1371/journal.pone.0083944 PMC3885544

